# Start, Stop, Continue? The Benefit of Overlapping Intravenous Thrombolysis and Mechanical Thrombectomy: A Matched Case-control Analysis from the German Stroke Registry

**DOI:** 10.1007/s00062-022-01200-y

**Published:** 2022-07-26

**Authors:** Egon Burian, Dominik Sepp, Manuel Lehm, Kathleen Bernkopf, Silke Wunderlich, Isabelle Riederer, Christian Maegerlein, Anna Alegiani, Claus Zimmer, Tobias Boeckh-Behrens, GSR-ET Investigators

**Affiliations:** 1 Department of Diagnostic and Interventional Neuroradiology, Klinikum rechts der Isar, Technical University of Munich, Ismaninger Str. 22, 81675 Munich, Germany; 2 Department of Diagnostic and Interventional Radiology, Klinikum rechts der Isar, Technical University of Munich, Munich, Germany; 3 Department of Diagnostic and Interventional Radiology, Klinikum Bogenhausen, Teaching Hospital of the Technical University of Munich, Munich, Germany; 4 Department of Neurology, Klinikum rechts der Isar, Technical University of Munich, Munich, Germany; 5 Department of Neurology, University Medical Center Hamburg-Eppendorf, Hamburg, Germany

**Keywords:** Acute ischemic stroke, Alteplase, Brain infarction, Anterior circulation, Brain revascularization

## Abstract

**Objective:**

Here we compare the procedural and clinical outcome of patients undergoing thrombectomy with running thrombolysis to matched controls with completed intravenous therapy and an only marginally overlapping activity.

**Methods:**

Patients from 25 sites in Germany were included, who presented with an acute ischemic stroke. Patients’ baseline characteristics (including ASPECTS, NIHSS and mRS), grade of reperfusion, and functional outcome 24 h and at day 90 after intervention were extracted from the German Stroke Registry (*n* = 2566). In a case-control design we stepwise matched the groups due to age, sex and time to groin puncture and time to flow restoration.

**Results:**

In the initial cohort (overlap group *n* = 864, control group *n* = 1702) reperfusion status (median TICI in overlap group vs. control group: 3 vs. 2b), NIHSS after 24 h, early neurological improvement parameters, mRS at 24 h and at day 90 were significantly better in the overlap group (*p* < 0.001) with a similar risk of bleeding (2.9% vs. 2.4%) and death (18% vs. 22%). After adjustment mRS at day 90 still showed a trend for lower disability scores in the overlap group (3 IQR 1-5 vs. 3 IQR 1-6, *p* = 0.09). While comparable bleeding risk could be maintained (4% in both groups), there were significantly more deaths in the control group (18% vs. 30%, *p* = 0.006).

**Conclusion:**

The presented results support the approach of continuing and completing a simultaneous administration of intravenous thrombolysis during mechanical thrombectomy procedures.

## Introduction

For a long time, intravenous administration of alteplase was considered the only proven treatment option for large vessel occlusion (LVO) within a time window of 4.5 h from symptom onset [1]. Intravenous thrombolysis (IVT) was standard of care until five randomized clinical trials proved the superiority of a treatment strategy combining mechanical thrombectomy (MT) and IVT [2–6]. Recent investigations revealed an early recanalization rate after sole alteplase administration for treatment of LVO in 41% with a good clinical outcome in only 29% [7]. Hence, until today the combinatorial approach of alteplase administration and mechanical thrombectomy is still the standard of care in ischemic stroke treatment.

Although there is a plentitude of data on the efficacy of MT and IVT reflected in national and international treatment guidelines, the temporal treatment workflow is still a point of discussion [8, 9]. Neurointerventionalists’ heterogeneous approaches in time management of IVT and MT in LVO were revealed in a national survey covering 107 treatment sites in Germany [10]. Even if the majority of participants reported to continue IVT after initiation of MT, there was a considerable number of clinicians who generally stop IVT either before or after MT or they decide on a case by case basis. After successful recanalization, most neurointerventionalists report to continue with IVT on a case dependent basis, e.g. thrombolysis in cerebral infarction (TICI) grade [10]; however, a certain minority exists, who do not continue IVT mostly because of a fear of increasing the risk of intracranial hemorrhage. These findings mirror a certain insecurity in coordination of IVT and MT in acute ischemic stroke treatment and are indicative of a missing strategy in cases of potential time overlap. In the past, the safety of a running IVT administration during MT using a stent retriever has been shown descriptively; however, there are still no comparative data to enable drawing conclusions with respect to continuing or termination of IVT before MT [11]. Additionally, there is no evidence regarding the impact of time overlap of IVT and MT on procedural or functional outcomes.

The just recently published randomized CHOICE trial [12] provides preliminary evidence for a clinical benefit of the adjunctive use of intra-arterial alteplase after successful mechanical thrombectomy but did not directly address possible overlapping efficacy of intravenous alteplase, as it is standard of care in most stroke centers to date.

This subgroup analysis of the GSR-ET (German Stroke Registry–Endovascular Treatment) addresses these questions and aims at quantitating the effects of a time overlap of IVT and MT (≥ 10 min) on treatment outcome and procedural safety in a case-control study design.

## Material and Methods

### Study Population

In this study 6635 patients of the GSR-ET (July 2015 to December 2019; http://ClinicalTrials.gov Identifier: NCT03356392) were screened and out of these, 2567 patients were included. The GSR-ET is an ongoing, open-label, retrospective, multicenter registry of 25 sites in Germany collecting consecutive patients undergoing MT. A detailed description of the GSR-ET study design has been published before [13].

**Fig. 1:**
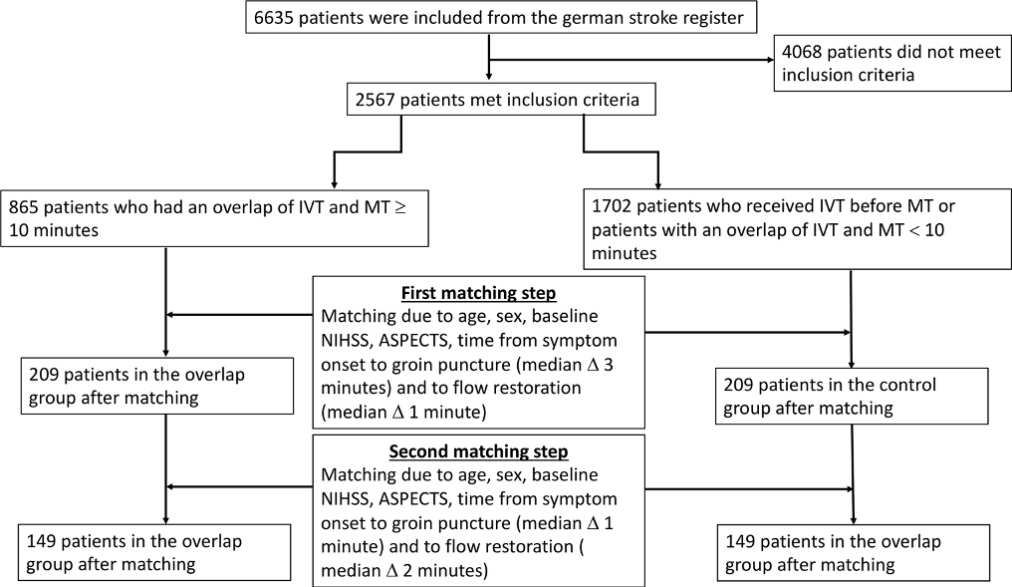
Flowchart showing the cohort composition and matching procedure. *ASPECTS* Alberta Stroke Program Early CT Score, *IVT* Intravenous thrombolysis, *MT* Mechanical thrombectomy, *NIHSS* National Institutes of Health Stroke Scale

A descriptive statistical comparison of the initial cohort is provided in Table 1. Study cohort composition is graphically described in Fig. 1.

**Table 1:** Demographic characteristics, baseline and follow up evaluation, workflow times and clinical outcome in all patients who met inclusion criteria

	IVT and MT overlap	IVT before MT over 50 min	*p*
*Demographic characteristics*			
Age (years)	76 IQR 65-83 (*n* = 864)	75 IQR 64-82 (*n* = 1702)	0.198
Sex, female No./total (%)	426/864 (49.2)	872/1702 (51.2)	0.356
*Clinical and imaging evaluation at presentation*			
Baseline pmRS	0 IQR 0-1 (*n* = 825)	0 IQR 0-1 (*n* = 1634)	0.163
Baseline NIHSS Admission	14 IQR 9-19 (*n* = 842)	15 IQR 10-18 (*n* = 1647)	0.817
Baseline ASPECTS	9 IQR 8-10 (*n* = 733) MW 8.8 ± 1.6	9 IQR 7-10 (*n* = 1390) M 8.4 ± 1.8	<0.001
ACA	2.2% *n* = 19/864	2% *n* = 34/1671	0.784
MCA	83.4% *n* = 721/864	90.1% *n* = 1507/1673	<0.001
MCAm1prox	32.8% *n* = 283/864	35.3% *n* = 590/1671	0.200
MCAm1dist	18.5% *n* = 160/864	21.8% *n* = 364/1671	0.054
MCAm2	22.6% *n* = 195/864	21.2% *n* = 355/1671	0.443
PCA	3.9% *n* = 34/864	2.1% *n* = 35/1671	0.007
*Workflow times*			
Time from SO to IVT	93 IQR 73-130 (*n* = 631)	90 IQR 70-120 (*n* = 1176)	0.03
Time from SO to FLR	172 IQR 140-215 (*n* = 556)	259 IQR 205-324 (*n* = 1054)	<0.001
Time from SO to GRO	125 IQR 105-163 (*n* = 621)	210 IQR 165-270 (*n* = 1227)	<0.001
*Clinical and imaging evaluation after intervention*			
Treat ae ICH	25 (2.9%) (*n* = 840)	41 (2.4%) (*n* = 1611)	1.0
24h NIHSS	6 IQR 2-16 (*n* = 743) MW 9.9 ± 9.9	10 IQR 4-18 (*n* = 1504) MW 12.1 ± 10.1	<0.001
BL-24 h NIHSS	5 (*n* = 743)	2.5 (*n* = 1498)	<0.001
24h NIHSS (0.1)	141/738 (19.1%)	150/1498 (10.0%)	<0.001
24h mRS	4 IQR 2-5 (*n* = 748) MW 3.5 ± 1.6	4 IQR 3-5 (*n* = 1543) MW 3.9 ± 1.4	<0.001
*Functional long-term outcome*			
D90 mRS	3 IQR 1-5 (*n* = 776) MW 2.8 ± 2.2	3 IQR 1-6 (*n* = 1458) MW 3.2 ± 2.2	<0.001
Death	163/865 (18%)	376/1702 (22%)	0.056
*Procedural outcome*			
Treat final TICI	3 (*n* = 850)	2b (*n* = 1679)	<0.001
0	5.8% (*n* = 49)	8.2% (*n* = 137)	–
1	1.5% (*n* = 13)	0.8% (*n* = 14)	–
2a	4.1% (*n* = 35)	6.3% (*n* = 105)	–
2b	32.6% (*n* = 277)	36.5% (*n* = 613)	–
3	56% (*n* = 476)	48.2% (*n* = 810)	–

*24h mRS* Modified Rankin Scale after 24 hours, *BL-24 h NIHSS* baseline NIHSS-NIHSS after 24 hours, *24h NIHSS (0.1)* NIHSS of 0 or 1 after 24 hours, *ACA* Anterior cerebral artery, *ASPECTS* Alberta Stroke Program Early CT Score, *D90 mRS* Modified Rankin Scale after 90 days, *FLR* flow restoration, *GRO* groin puncture, *IVT* Intravenous thrombolysis,  *MCA* Middle cerebral artery, *NIHSS* National Institutes of Health Stroke Scale, *PCA* Posterior cerebral artery, *pmRS* pretreatment modified Rankin Scale, *SO* symptom onset, *TICI* Thrombolysis in cerebral infarction, *Treat ae ICH* post treatment intracranial hemorrhage, *Treat final TICI* TICI after treatment

The main inclusion criteria for all cases were (1) the diagnosis of an acute ischemic stroke attributed to large vessel occlusion, (2) intravenous alteplase administration, (3) endovascular treatment, (4) a prestroke modified Rankin Scale (mRS) 0–3. At a minimum time overlap of 10 min for IVT and MT patients were assigned to the case group. In the control group IVT had an overlap of less than 10 min or was finished prior to MT.

An overlap time of at least 10 min of analogous initiation of MT and running IVT was chosen, as the median time from groin puncture to flow restoration was around 50 min and the second half-life of alteplase for deeper tissue compartments is 40 min [14]. Thus, a sufficient pharmacological effect of the administered thrombolysis is highly probable during the endovascular procedure in this subgroup.

The initial case groups could not be well balanced especially regarding time-dependent decisive outcome affecting parameters. Therefore, we applied a matched case-control model performing the following steps to obtain two comparable case groups:

In the first step we matched the two groups with regard to age (max. age difference 5 years), sex (equal sex distribution), baseline NIHSS (National Institutes of Health Stroke Scale) (max. difference 5 points), baseline ASPECTS (Alberta Stroke Program Early CT Score) (max. difference 1 point), time from symptom onset to groin puncture (max. difference of 10 min) and time from symptom onset to flow restoration (max. difference of 10 min).

To obtain an even more accurate comparability, we further tightened the accepted time differences with the consequence of a diminished case number in each group in a second matching step. Thus, time from symptom onset to groin puncture and time from symptom onset to flow restoration were further reduced to a max. difference of 5 min each.

The study was conducted in accordance with the Declaration of Helsinki.

## Study Endpoints

The primary functional outcome parameter was mRS at 90-day follow-up. Further surrogate markers were NIHSS at 24 h after MT/IVT, ∆NIHSS baseline/NIHSS after 24 h and NIHSS of ≤ 1 after 24 hours (surrogate parameters for early neurological improvement). Major neurological improvement was defined by a  ≥ 8-point improvement in NIHSS score or an NIHSS score of 0 or 1 at 24 h [15].

The angiographic endpoint was the successful recanalization of the occluded target vessel assessed after each intervention on digital subtraction angiography with the Thrombolysis in Cerebral Infarction (TICI) score ≥ 2b. Occurrence of symptomatic intracranial hemorrhage and death were included as adverse events associated with MT/IVT.

## Statistical Analysis

Univariate distribution of metric variables is described by median and IQR. For categorical data, absolute and relative frequencies are given. Standard descriptive statistics were used for all presented data.

Initially, the Kolmogorov–Smirnov test was used to test data distribution in regard of normality.

Baseline characteristics were compared by outcome performing Fisher’s exact test for categorical variables, Mann–Whitney U test (non-normally distributed data), and the unpaired Student’s t test (normally distributed data) for continuous variables. In a case-control approach we excluded age, sex, baseline NIHSS, ASPECTS and time to groin puncture and time to flow restoration as potential confounding factors. The therapy group was defined with regard to start of IVT and groin puncture (maximum of 10 min difference). In the control group IVT was started more than 10 min before groin puncture. All patients were selected from the GSR-ET using a case-control approach of the Statistical Package for Social Science (SPSS 25.0 for Windows, SPSS, Inc, Chicago, IL, USA).

Significance level was set at α = 0.05. All statistical analyses were carried out using SPSS software (IBM SPSS Statistics, version 2 5.0; IBM, Armonk, NY, USA).

## Results

A total of 864 patients fulfilled the criteria for the overlap group and 1702 patients were in the control group. Median age was 76 years in the overlap group (IQR 65–83 years), and 49.2% (426 of 864) were women. On hospital admission, median National Institutes of Health Stroke Scale (NIHSS) score was 14 (IQR, 9–19) in the overlap group and 15 (IQR, 10–18) in the control group. Alberta Stroke Program Early CT Score (ASPECTS) was 9 in both groups (IQR overlap group, 8–10; IQR control group: 7–10). Most occlusions in the overlap group were located in the MCA (83.4%; 721 of 864), 2.2% cases showed an occlusion of the ACA and 3.9% of the PCA. Within this initial cohort significant differences could be detected in time to groin puncture and time to flow restoration amongst others (Table 1). In the following steps we gradually reduced each group with regard to an equal age and sex distribution, similar ASPECTS and NIHSS admission scores and a comparable time to groin puncture and time to flow restoration.

### First Matching Step

After stepwise fitting the data to a case-control model, there were no statistically significant differences between the two groups regarding age, sex, baseline ASPECTS and NIHSS, time from symptom onset to groin puncture and time from symptom onset to flow restoration. In the first approach to homogenize the two groups the differing workflow times were matched to a delta of 10 min with regard to time from symptom onset to flow restoration and time from symptom onset to groin puncture (Table 2). In this cohort the overlap group showed higher complete reperfusion levels (TICI 3: 57.9% vs. 47%), while successful recanalization (TICI ≥ 2b) was achieved in 90.3% (control: 86.7%).

**Table 2:** Demographic characteristics, baseline and follow-up evaluation, workflow times and outcome in patients after first matching step

	IVT and MT overlap	IVT before MT over 50 min	*p*
*Demographic characteristics*			
Age (years)	76 IQR 67-82 (*n* = 209)	76 IQR 67-82 (*n* = 209)	0.963
Sex, female No./total (%)	108/209 (51.7)	108/209 (51.7)	1
*Clinical and imaging evaluation at presentation*			
Baseline pmRS	0 IQR 0-1 (*n* = 203)	0 IQR 0-1 (*n* = 207)	0.300
Baseline NIHSS Admission	14 IQR 9-18 (*n* = 207)	14 IQR 9-18 (*n* = 207)	1
Baseline ASPECTS	9 IQR 8-10 (*n* = 189)	9 IQR 8-10 (*n* = 184)	0.868
*Workflow times*			
Time from SO to IVT	120 IQR 90-155 (*n* = 203)	70 IQR 55-85 (*n* = 202)	<0.001
Time from SO to FLR	199 IQR 163-236 (*n* = 195)	198 IQR 170-238 (*n* = 181)	0.708
Time from SO to GRO	149 IQR 125-184 (*n* = 209)	152 IQR 127-180 (*n* = 209)	0.628
*Clinical and imaging evaluation after intervention*			
Treat ae ICH	8 (3.8%) (*n* = 209)	7 (3.3%) (*n* = 209)	1.0
24h NIHSS	7 IQR 2-15 (*n* = 189) MW 9.9 ± 9.4	8 IQR 3-16 (*n* = 194) MW 10.3 ± 8.6	0.384
BL-24 h NIHSS	4 IQR 0-9 (*n* = 188)	3 IQR 0-8 (*n* = 193)	0.118
24h NIHSS (0.1)	32/209 (15.3%)	32/209 (15.3%)	–
24h mRS	4 IQR 2-5 (*n* = 190) MW 3.6 ± 1.6	4 IQR 2-5 (*n* = 197) MW 3.6 ± 1.6	0.950
*Functional long-term outcome*			
D90 mRS	3 IQR 1-5 (*n* = 184) MW 2.8 ± 2.2	3 IQR 1-6 (*n* = 192) MW 3.2 ± 2.2	0.052
Death	29/184 (15.8%)	51/192 (26.6%)	0.013
*Procedural outcome*			
Treat final TICI	3 (*n* = 207)	2b (*n* = 204)	0.026
0	2.6% (*n* = 6)	5.9% (*n* = 12)	–
1	2.4% (*n* = 5)	0.9% (*n* = 2)	–
2a	4.3% (*n* = 9)	6.4% (*n* = 13)	–
2b	32.4% (*n* = 67)	39.7% (*n* = 81)	–
3	57.9% (*n* = 120)	47.0% (*n* = 96)	–

*24h mRS* Modified Rankin Scale after 24 hours, *BL-24 h NIHSS* baseline NIHSS-NIHSS after 24 hours, *24h NIHSS (0.1)* NIHSS of 0 or 1 after 24 hours, *ACA* Anterior cerebral artery, *ASPECTS* Alberta Stroke Program Early CT Score, *D90 mRS* Modified Rankin Scale after 90 days, *FLR* flow restoration, *GRO* groin puncture, *IVT* Intravenous thrombolysis,  *MCA* Middle cerebral artery, *NIHSS* National Institutes of Health Stroke Scale, *PCA* Posterior cerebral artery,  *pmRS* pretreatment modified Rankin Scale, *SO* symptom onset, *TICI* Thrombolysis in cerebral infarction,  *Treat ae ICH* post treatment intracranial hemorrhage, *Treat final TICI* TICI after treatment

Further, after the first adjustment step the overlap group revealed nearly significantly better functional outcome with regard to D90 mRS (overlap group: mRS 3 IQR 1–5; control group: mRS 3 IQR 1–6, *p* = 0.052). While in the overlap group 15.8% of patients died after treatment, over one quarter of patients died in the control group (26.6%, *p* = 0.013). No significant differences could be detected in percentages of patients with best functional outcome at day 90 (mRS 0) (*p* = 0.192).

### Second Matching Step

After performing the second matching step, median time from onset to groin puncture was 154 min (IQR, 128–187 min) and median time to flow restoration was 203 min (IQR, 164–244 min) in the overlap group (control: time to groin puncture: 155 min IQR, 127–184 min; time to flow restoration was 201 min IQR, 177–244 min) (Table 3).

**Table 3:** Demographic characteristics, baseline and follow up evaluation, workflow times and outcome in patients after second matching step

	IVT and MT overlap	IVT before MT over 50 min	*p*
*Demographic characteristics*			
Age (years)	76 IQR 68-82 (*n* = 149)	77 IQR 68-82 (*n* = 149)	0.685
Sex, female No./total (%)	82/149 (55)	82/149 (55)	1
*Clinical and imaging evaluation at presentation*			
Baseline pmRS	0 IQR 0-1 (*n* = 143)	0 IQR 0-1 (*n* = 147)	0.364
Baseline NIHSS Admission	14 IQR 9-17 (*n* = 148)	14 IQR 9-17 (*n* = 148)	1
Baseline ASPECTS	9 IQR 8-10 (*n* = 134)	9 IQR 8-10 (*n* = 132)	0.495
*Workflow times*			
Time from SO to IVT	120 IQR 90-152 (*n* = 145)	70 IQR 55-90 (*n* = 144)	<0.001
Time from SO to FLR	203 IQR 164-244 (*n* = 135)	201 IQR 177-244 (*n* = 131)	0.579
Time from SO to GRO	154 IQR 128-187 (*n* = 149)	155 IQR 127-184 (*n* = 149)	0.957
*Clinical and imaging evaluation after intervention*			
Treat ae ICH	6 (4%) (*n* = 149)	6 (4%) (*n* = 149)	–
24h NIHSS	8 IQR 2-16 (*n* = 131) MW 10.7 ± 10.2	8 IQR 4-18 (*n* = 137) MW 10.4 ± 8.5	0.733
BL-24 h NIHSS	4 IQR 0-7 (*n* = 131)	4 IQR −1-8 (*n* = 136)	0.834
24h NIHSS (0.1)	23/131 (17.5%)	22/137 (16.1%)	–
24h mRS	4 IQR 2-5 (*n* = 133) MW 3.6 ± 1.6	4 IQR 2-5 (*n* = 140) MW 3.6 ± 1.6	0.648
*Functional long-term outcome*			
D90 mRS	3 IQR 1-5 (*n* = 132) MW 2.9 ± 2.2	3 IQR 1-6 (*n* = 140) MW 3.4 ± 2.3	0.090
Death	25/132 (18.9%)	43/140 (30.7%)	0.006
*Procedural outcome*			
Treat final TICI	3 (*n* = 148)	2b (*n* = 147)	0.238
0	3.3% (*n* = 5)	6.8% (*n* = 10)	–
1	3.3% (*n* = 5)	0.6% (*n* = 1)	–
2a	6.1% (*n* = 9)	6.8% (*n* = 10)	–
2b	34.5% (*n* = 51)	39.5% (*n* = 58)	–
3	52.7% (*n* = 78)	46.3% (*n* = 68)	–

*24h mRS* Modified Rankin Scale after 24 hours, *BL-24 h NIHSS *baseline NIHSS- NIHSS after 24 hours, *24h NIHSS (0.1)* NIHSS of 0 or 1 after 24 hours, *ACA *Anterior cerebral artery, *ASPECTS* Alberta Stroke Program Early CT Score, *D90 mRS* Modified Rankin Scale after 90 days, *FLR* flow restoration, *GRO* groin puncture, *IVT* Intravenous thrombolysis, *MCA* Middle cerebral artery, *NIHSS* National Institutes of Health Stroke Scale, *PCA* Posterior cerebral artery,  *pmRS* pretreatment modified Rankin Scale, *SO* symptom onset, *TICI* Thrombolysis in cerebral infarction, *Treat ae ICH* post treatment intracranial hemorrhage, *Treat final TICI* TICI after treatment

In the overlap group 52.7% reached a postinterventional TICI 3 (control: 46.3%). Successful recanalization was achieved in 87.2% (129 of 148) in the overlap group and in 85.8% (126 of 147) in the control group. In 5 patients of the overlap group recanalization was considered unsuccessful (TICI 0), compared with 10 patients in the control group (Table 3). In both groups 6 patients were diagnosed with intracranial hemorrhage in posttreatment imaging (4%) (Table 3). A preserved good functional outcome could be shown in both groups (overlap group: mRS 3, IQR 1–5; control group: mRS 3, IQR 1–6). After the second matching step a statistical tendency towards a better outcome in the overlap group could be detected (*p* = 0.09).

Neither NIHSS after 24 h nor ∆NIHSS baseline/NIHSS after 24 h showed significant differences in the two groups. Of note, posttreatment mortality was 18% in the overlap group and 30% in the control group (*p* = 0.006) (Table 3). Comparing both groups no significant differences could be detected with regard to the most favorable mRS outcome of 0 (*p* = 0.148).

**Fig. 2:**
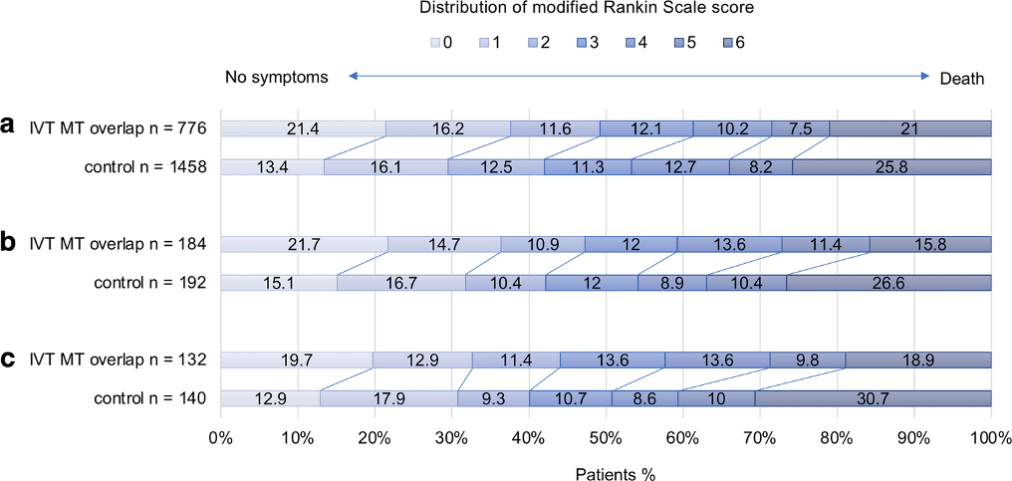
In this shift analysis the distribution of modified Rankin Scale score at day 90 is shown. **a** Includes the initial unmatched cohort, **b** cohort after first matching step, **c** cohort after second matching step. *IVT* Intravenous thrombolysis, *MT* Mechanical thrombectomy

Functional outcome parameters (mRS after 90 days) for the initial cohort as well as after stepwise matching are shown in Fig. 2.

## Discussion

The combination of IVT and MT as a matter of principle is still common sense and widely accepted in the stroke community. Recently, there are several randomized controlled trials questioning this approach and first results show a non-inferiority of EVT alone vs. a combination therapy [16–18]. Podlasek et al. concisely summarized the combined trial data of six studies including SWIFT-DIRECT and DIRECT-SAFE, showing a clinical non-inferiority of direct mechanical thrombectomy to bridging therapy [19] but significantly better technical success rates in the MT/IVT groups; however, there is still a paucity of evidence concerning the concomitant use of IVT and MT with overlapping lysis activity during the MT procedure. As there are no guidelines on the temporal orchestration of IVT and MT, a certain insecurity has spread among neurointerventionalists and neurologists whether to stop or to continue alteplase administration before initiation or after completion of the thrombectomy procedure [10]. This ambiguity is based on concerns regarding iatrogenically increasing the risk of symptomatic intracranial hemorrhage or thrombus fragmentation if MT is performed under concomitant unmitigated potency of IVT, which leads to a discussion of principles [10, 20–22]. Summarizing the results of the presented study several key aspects and major concerns regarding the execution of IVT are touched.

First, the procedural as well as the functional outcome in patients undergoing overlapping IVT and MT does not differ significantly after matching of the two groups was performed. It rather seems that patients in the overlap group might benefit from parallel IVT administration as a tendency towards lower D90 mRS scores (*p* = 0.09) than in the control group could be revealed. Interestingly, unsuccessful recanalization was twice as high in the control group as in the overlap group (TICI 0: 3.3% vs. 6.8%). Second, the risk of postinterventional intracranial hemorrhage was not increased in the overlap group (4% in both groups after adjustment). Third, postinterventional mortality was not increased when performing overlapping IVT and MT. Quite the contrary, comparing the two groups, 18% of patients in the overlap group died after thrombectomy, whereas nearly one third of patients in the control group died (30%, *p* = 0.006). Taken together, the results of this study suggest that there is no need for stopping IVT immediately before starting or after having completed the MT procedure in patients with running alteplase administration. These results seem to be in line with the recently presented preliminary data from the SWIFT-DIRECT trial [24], as the investigated lysis group in this trial mainly consisted of mothership patients with short procedure times, making an overlapping lysis activity in this group probable. This actually raises the additional question if the positive trial results may be partially based on the positive effect of an overlapping lysis effect, and the results may have been less clear, if also transfer patients with no overlapping activity would have been included.

In addition, our results strongly support the results of the most recently published CHOICE trial [12], which shows an additional benefit of the adjunctive use of intra-arterial alteplase after successful recanalization. Especially the remarkable effect on reducing mortality and the similarity of the mRS shift analyses are surprisingly consistent in both studies. In this context, our results seem to add evidence to the question whether the intravenous approach might be sufficient compared with an additional intra-arterial application of alteplase.

There are several possible reasons for a tendentially better functional outcome in the overlap group compared to the control group. During the closer analysis of the differences in the mRS values, it became clear that very similar to the results of the CHOICE trial, the differences in favor of the overlap group are mainly based on the switch of patients from mRS 1 to 0 and to a lesser extent, from 3 and 4 to 6 (see Fig. 2). The first effect is probably explainable by the lysis of persistent micro-occlusions in the capillary vasculature or very peripheral occlusions due to thrombus fragments after successful MT of the large vessel occlusion, leading to a higher percentage of fully asymptomatic patients. Also, more frequently occurring thrombus migration in the control group to mechanically inaccessible regions due to longer impact times of the lysis might contribute to this effect. This also might explain the results of fewer postprocedural angiograms classified as TICI 3 in all controls. The second effect might be due to the same effect in the opposite group of patients suffering from a severe stroke despite successful recanalization, who develop without IVT an even bigger malignant infarction with a higher percentage of deceased patients.

To the best of our knowledge, besides the CHOICE trial addressing an additional intra-arterial approach, there are no published data available investigating the problem we focused on in the actual study. One recent publication, also from the GSR investigators, found a better outcome in patients with inhouse bridging lysis (which should in many cases be overlapping to MT) compared to patients without lysis therapy [23, 24]. But, although these data might also support the notion of the safety of concomitant lysis, their work does not address the same topic, as they compared lysis vs. non-lysis patients and not unmitigated active lysis vs. previously iv-treated patients. Additionally, by having access to much more patient datasets, our approach of a matched pair analysis became possible.

The main strength of our study is the large patient number of a real-world population, which enabled a very exact matching of the groups with still considerable patient numbers. Second, we provide additional evidence for applying the direct clinical consequence of not stopping running thrombolysis, before, during or after the MT procedure (obviously, unless no other causes demand this).

However, it has to be considered, that the presented results are dependent on the organizational and infrastructural characteristics of the German stroke system regarding localization of primary and tertiary stroke centers and the associated infrastructure as well as transportation times. While mechanical thrombectomy can only be performed in tertiary care institutions, thrombolysis is frequently initiated in peripheral hospitals leading to a limited number of patients where overlapping thrombolytic activity can be achieved. This is even more true in regions with more centralized organizational structures, where secondary transport is even more common and transportation times might be longer.

Besides this our study has certain limitations. First of all, this is a retrospective study with all shortcomings associated with this conceptual design, resulting in a certain selection bias; however, applying the described matching steps (including baseline NIHSS as a decisive matching parameter), we tried to minimize this bias as far as possible. Furthermore, considering the observational character of our analysis, remaining confounding factors cannot be excluded. Additionally, all collected clinical and imaging data are prone to subjectivity due to the multicenter study design without independent re-evaluation.

## Conclusion

In conclusion, the presented multicenter study provides evidence for the approach of not stopping running thrombolysis before, during or after the MT procedure in acute stroke treatment, unless no other inevitable causes demand this. The data reveal a tendency for better functional outcome, with comparable risk for intracranial hemorrhage and lower postinterventional mortality in the overlap group. The results strongly support the recently published data of a possible benefit of additional intra-arterial alteplase administration after successful MT. These results may have immediate practical implications for acute treatment strategies.
